# Assessment of Plant Growth Promoting and Abiotic Stress Tolerance Properties of Wheat Endophytic Fungi

**DOI:** 10.1155/2019/6105865

**Published:** 2019-03-27

**Authors:** Farhana Alam Ripa, Wei-dong Cao, Shuai Tong, Jian-guang Sun

**Affiliations:** Key Laboratory of Microbial Resources, Ministry of Agriculture/Institute of Agricultural Resources and Regional Planning, Chinese Academy of Agricultural Sciences, Beijing 100081, China

## Abstract

The aims of the present work were to isolate and characterize fungal endophytic communities associated with healthy wheat (*Triticum aestivum L.*) plants, collected from the North China. Segregated endophytes were screened for their PGP traits, abiotic stresses (heavy metals, salinity, drought, and temperature), and antibiotic sensitivity. A total of 16 endophytic fungi were isolated using the culture-dependent approach from different tissue parts of wheat plants. Based upon their internal transcribed spacer (ITS) rDNA gene sequencing, 15 out of 16 isolates were selected for further analysis. In the contemporary investigation, a number of the tested endophytes exhibited fairly good 1-aminocyclopropane-1-carboxylic acid deaminase (ACCD) (0.03±0.011 to 1.43±0.01 *µ*mol *α*-KB mg^−1^ protein hr^−1^), indole acetic acid (IAA) (1.125±0.04 to36.12±0.004*µ*gml^−1^), and phosphate solubilizing index (PSI) (2.08±0.03to5.16±0.36) activities. More than 30% isolates gave positive result for siderophore and ammonia tests, whereas all exhibited catalase activity but only 2 (582PDA1 and 582PDA11) produced hydrogen cyanide.* Trichoderma* strains showed salt, heavy metals, and drought tolerance at high levels and also exhibited resistance to all the tested antibiotics. Strain 582PDA4 was found to be the most temperature (55°C) tolerant isolate. The findings of this study indicated that the microbial endophytes isolated from wheat plants possessing a crucial function to improve plant growth could be utilized as biofertilizers or bioagents to establish a sustainable crop production system.

## 1. Introduction

Globally wheat is considered as one of the major cereal crops. According to Food and Agricultural Organization (FAO) of the United Nations, its demand will be amplified up to 746 million tons by 2020 [1]. This raise in production desires to be accomplished in spite of the budding challenges to modern agriculture as well as precincts in the application of pesticides [2], concerns about the accessibility and environmental impact of fertilizer inputs [3], and the potential harmful impacts of climate alteration on wheat yields and disease spectrum [4]. Cultivation of high yielding varieties of crops, rigorous cropping system, and unevenness use of chemical fertilizers are the core factors which develop nutrient discrepancy in soil, squat yield, shrinkage of soil fertility, and stumpy quality of food. Hence, it turns to a severe problem to develop sustainable tactics for mitigation of unfavorable effect of intensive practices used by peasants [5]. The question of primary production sustainability is heightened more for wheat. Agricultural scientists around the planet are working round the clock to look for novel options to enhance agricultural productivity, sustainability, but it undoubtedly represents an immense challenge for them. The use of beneficial microbial symbionts of plants with the objective of improving agricultural productivity is one of the most important sustainable practices [6]. Concerning to reduce the harmful effects of the conventional methods of agriculture; innovative schemes based on microbial inoculation are currently gaining more attention. Plants and microorganisms form a symbiotic alliance with reimbursement for both cohorts. Additionally, plant-microbe symbiosis influences plant growth and health which efficiently ameliorates agricultural traits and improve soil quality and nutrient cycling [7–9].

Normally a number of microbes are found to acquire nutrients for their continued existence through interaction with plants which might be impartial, harmful (parasitism), or beneficial (mutualism or symbiosis) to the host [10, 11]. Microbes that inhabit within the plant tissues devoid of doing substantive damage or acquiring remuneration other than securing their residency are considered as ‘endophytes'. Plants have been blessed by nature with diversified population of endophytic microorganisms including beneficial bacteria, fungi, and actinomycetes. They spend their entire or part of the life cycle living inside the plant causing no visual symptoms of disease [12]. Organization of such useful plant coupled microbes is continuously gaining attention among scientific community and at the view point of industries due to their aptitude to advance plant quality and growth [13, 14].

Species of fungi that reside within living plant tissue without causing symptoms of disease in their host are known as fungal endophytes [15]. They are the major members of endophytic population that dwell entirely within plant tissues and may be allied with roots, stems, and/or leaves. Every plant harbor at least one or more endophytic fungi in the universe [16, 17]. In recent years, they have been extensively studied in diverse geographic and climatic regions and were found to be ubiquitous inside plant tissues and rich in species diversity [18–21]. Researchers found their precious roles in nutrients supply, environment acclimatization, biotic and abiotic stresses protection, growth promotion and enhancing community biodiversity of host plants [22–26]. They can also act as warden against predators [27] and contestants of microbial pathogens [28]. Previous reports showed that many grass species indicated vegetative growth improvement in the presence of their fungal symbionts that have been primarily credited to enlarge plant fitness [29, 30]. Notwithstanding, late examinations have demonstrated that plant growth promotion might be attributed to the discharge of plant growth advancing secondary metabolites (gibberellins, auxins, cytokinin) by the endophytic fungi in the rhizosphere [29]. Literature survey additionally demonstrated that Plant Growth Promoting Fungi (PGPF) maintain plant growth through the generation of a number of significant enzymes like ACCD, urease, catalase, etc., phosphate solubilization, siderophore and IAA formation and antagonism to phytopathogens and take a crucial part in plant growth [30–33]. Earlier reports showed that antibiotic resistant PGP endophytes could be a good source of biocontrol agents [34, 35].

Although formerly a number of researches were conducted on wheat endophytic fungi [36–38], but till now no report has been found on their PGP traits along with their resistance pattern to abiotic stresses and antibiotics. Therefore we designed this study to evaluate the plant growth promoting traits along with abiotic stress tolerance and antibiotics resistance properties of wheat endophytic fungi which would be a potential source of biofertilizers in a sustainable organic crop production system in the foreseeable future.

## 2. Material and Methods

### 2.1. Plant Sampling and Isolation of Endophytic Fungus

Wheat plant samples were collected from Henan (Jinshui District-34° 46′ 22.59^″^ N, 113° 43′ 9.62^″^ E, altitude 100 meters), Shandong (Dezhou-37°26′N, 116°16′E, altitude 50 meters), and Hebei (Anxin county-38°55′N 115°56′E, altitude 80 meters) provinces of Northern China on 20th April 2017 and processed in the laboratory within 24 h. They were checked carefully for any disease symptoms or superficial damage and were washed thoroughly in running tap water to eradicate the superficial dirt of plant parts. In the wake of washing, the samples were separated into leaves, stems, and roots. Endophytic fungi were isolated from the sound and asymptomatic roots, leaves, and stems of the experimented plant samples based on the published protocols [38, 39]. Competence of the surface sterilization strategy was confirmed by engrave technique [14]. The sterilized plant parts were placed aseptically on potato dextrose agar (PDA) medium and incubated at room temperature for 5-7 d. The fungi appeared from the edges of the inoculated parts were isolated and identified and pure cultures were maintained on potato dextrose agar slants.

### 2.2. Molecular Identification and Phylogenetic Analysis of the Isolated Endophytic Fungus

**Figure 1:**
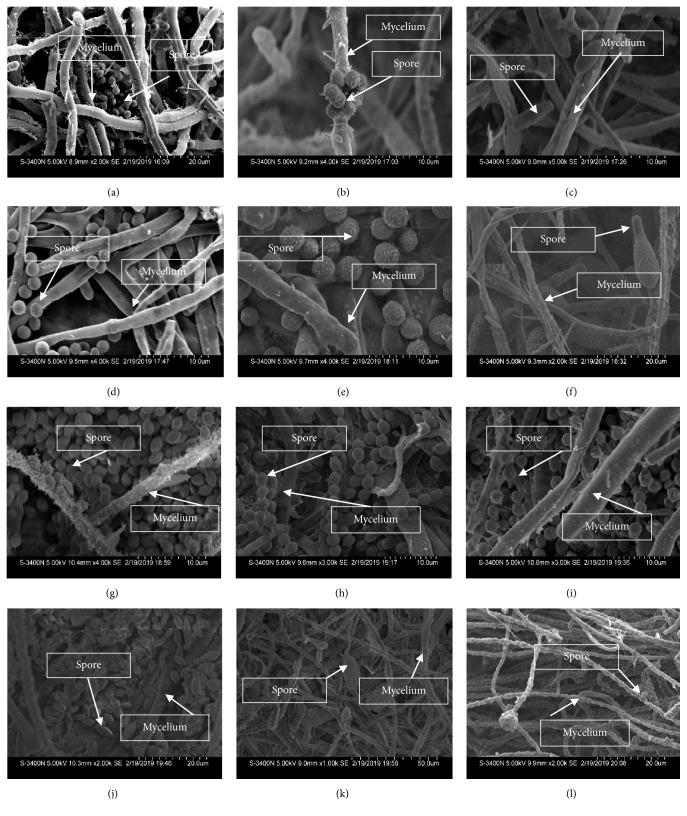
Electromicrographs of wheat fungi in 11 days old PDA plates ((a)* T. aureoviride*; (b)* T. harzianum*; (c)* F. proliferatum*; (d)* P. janthinellum*; (e)* A. flavus*; (f)* A. tenuissima*; (g)* T. funiculosus*; (h)* P. aurantiogriseum*; (i)* A. stellatus*; (j)* C. cladosporioides*; (k)* A. alternate*; (l)* F. equiseti*).

Molecular identification was carried out on the basis of fungal internal transcribed spacer (ITS) rDNA rejoins amplification and sequence analyses. Genomic DNA was extracted with Tiangen Fungus DNA kit (Biotech, Beijing, Co., Ltd.) according to the manufacturer's instructions. Amplification of fungal ITS region was carried out with ITS1 and ITS4 as forward and reverse primer, respectively [40]. The PCR products were checked for the expected size on 1% agarose gel and were sequenced by Sangon sequence service provider (Sangon Inc. Beijing, China). The nucleotide sequences were compared against nucleotide databases using the NCBI BLASTn program to identify the closest known taxa. The ITS-rDNA gene along with their closest homology sequences were aligned using multiple sequence alignment program CLUSTALW algorithm implemented in MEGA 7 software with default parameters [41]. Phylogenetic tree was constructed with neighbor-joining method by MEGA7 program. As a statistical support, bootstrap replications (1000) were used for the nodes in the phylogenetic tree. Based on molecular identification report, 15 fungi were chosen for further study. To take scanning electron micrographs of wheat fungi, they were dehydrated in a graded mounting ethanolic series, critical point-dried (CO_2_), coated with a thin layer of gold, and observed by means of a scanning electron microscope (Hitachi S-3400N). Some SEM images of wheat fungi are given in Figure 1.

### 2.3. Evaluation of Some Important PGP Properties

All isolates were screened for a wide range of vital PGP properties. ACCD potentiality of the fungal isolates were checked by both qualitative and quantitative ways using Dworkin and Foster (DF) minimal salts media [42] supplemented with 3 mM ACC as the sole nitrogen source [43–45]. Final ACCD activity was expressed in nanomol *α*-ketobutyrate (*α*-KB) mg^−1^ protein h^−1^. IAA production ability was tested using Salkowski's reagent as delineated by Acuña et al. [46]. Siderophore manufacturing capability of the selected endophytes was studied by universal Chrome Azurol S (CAS) agar plate method [47]. Phosphate solubilization property of all fungal isolates was tested by bromophenol blue (0.01-0.001 mg liter^−1^) added to Pikovskaya's agar media [48]. Ammonia (NH_3_), urease, and catalase production aptitude were studied as in previously described methods [49]. Cyanogenesis (hydrogen cyanide-HCN production) property of the fungal isolates was checked with picric acid soaked filter paper containing PDA plates [50].

### 2.4. Abiotic Stress Tolerance

A number of abiotic stress tolerance tests (salt, heavy metals, drought, and temperature) were conducted on different levels with fresh cultures of the isolated endophytic fungi. The intrinsic salinity resistance test cultures were checked by observing their growth on PDA media amended with different concentration (2.5-10% w/v) of sodium chloride (NaCl) at 28°C for 5 d. Heavy metal tolerance stress was assayed by growing the fungi on freshly prepared PDA plates amended with a variety of soluble heavy metal salts (nickel-Ni, lead-Pb, copper-Cu, cadmium-Cd and cobalt-Co) in different concentrations ranging from 50 to 300 *µ*g ml^−1^ at 28°C for 5 d [43]. Tolerance to drought stress was evaluated by Leo Daniel et al. method [50] with 10-40% polyethylene glycol (PEG 6000 Da) amended PDA plates. Temperature resistance was assessed by incubating fungi at diverse temperature regime (namely, 5, 15, 25, 35, 45, and 55°C) for 5 d [51].

### 2.5. Antibiotic Sensitivity Test

Antibiotic resistance is considered as one of the parameters to search for efficient biological control agents [52] and previous literature showed that this property can initiate plant growth to a certain extent [34, 35, 53]. Here, we have evaluated antibiotic sensitivity of the isolated strains against nystatin (10 *µ*g), ketoconazole (50 *µ*g), and itraconazole (30*µ*g) soaked discs (6 mm diameter) by Kirby Bauer disc-diffusion assay. Depending on the inhibition zone, organisms were grouped as resistant or sensitive according to the published literatures [54, 55]. Each experiment was repeated thrice for each fungus.

### 2.6. Statistical Analysis

Microsoft Excel 2013 was used to accomplish statistical analysis. The phylogenetic tree was constructed with MEGA7 software. All the experiments were carried out in triplicate. Means and standard deviations were estimated and applied.

## 3. Results

In the contemporary study, we have assayed a number of PGP traits of the isolated fungi which might play a crucial role on plant growth in both direct and indirect manners (all results are given in Table 2). In ACCD screening, 11 fungi passed qualitative test and were chosen for quantitative assay, among them 9 endophytic fungal strains exhibited negligible to moderate enzyme activity (0.03±0.011 to 1.43±0.01*µ*mol *α*-KB mg^−1^ protein hr^−1^). Nine fungi gave positive response for IAA with isolate 582PDA4 being the top producer (21.125±0.009*µ*gml^−1^), which proved them to be plant growth promoters. Siderophore producing aptitude was found for only 3 (581PDA1, 582PDA6, and 582PDA7) isolates in variable extents as evidenced by the formation of orange halo around the colony in CAS plate. Among the 15 fungi, 11 strains were marked to be phosphate solubilizers showing apparent halo zones around their colonies on Pikovskaya's agar medium in variable phosphate solubilizing index (PSI) scale (2.08±0.03 to 5.16±0.36). HCN test was positive for only 2 isolates, while NH_3_ was produced by around 34% of the tested microbes. For urease and catalase tests, we have found that none of the isolates possess urease enzyme but all of them exhibited catalase activity.

The abiotic stress tolerance capabilities of the isolates were checked by inspection of their growth in different levels of salt, heavy metals, drought, and temperature. Utmost growth tolerance was revealed by 582PDA6 and 582PDA7 under salt stress condition whereas almost all the fungi were able to grow at 5% of salt (except 582PDA5 and 582PDA11) which proved them to be supportive for saline prone agricultural areas (Table 3). Again, the fungal endophytes exhibited a speckled level of resistance to the tested heavy metals. Strains 582PDA6 and 582PDA7 were found to be resistant against all the tested concentrations of heavy metals while other isolates were resistant to different concentrations of the metals in different levels (Table 4). This study revealed that all the selected wheat endophytic fungi are able to resist drought in variable range. Profuse sporulation was observed in presence of 10-20% PEG concentrations for all strains, whereas at 35% PEG concentration sporulation was completely abolished for all strains. Highest drought tolerance potency was observed by strain 582PDA6 (Table 5). Here we also studied the effect of different range of temperature on the tested fungal isolates and found the optimum growth temperature of maximum strains is 25°C. The least and utmost temperature tolerated by the isolates were recorded as 5°C (581PDA1) and 55°C (582PDA4), respectively (Table 6). So we may say that these microbes can help to tolerate temperature stress to certain extent.

Antibiotic resistance pattern of all the experimented strains varied from antibiotic to antibiotic. Four fungal strains (581PDA3, 581PDA4, 582PDA6, and 582PDA7) were found to be resistant against all three antibiotics tested while three fungi, 581PDA2, 581PDA5, and 581PDA7, were sensitive to all three antibiotics (Table 7).

## 4. Discussion

**Figure 2:**
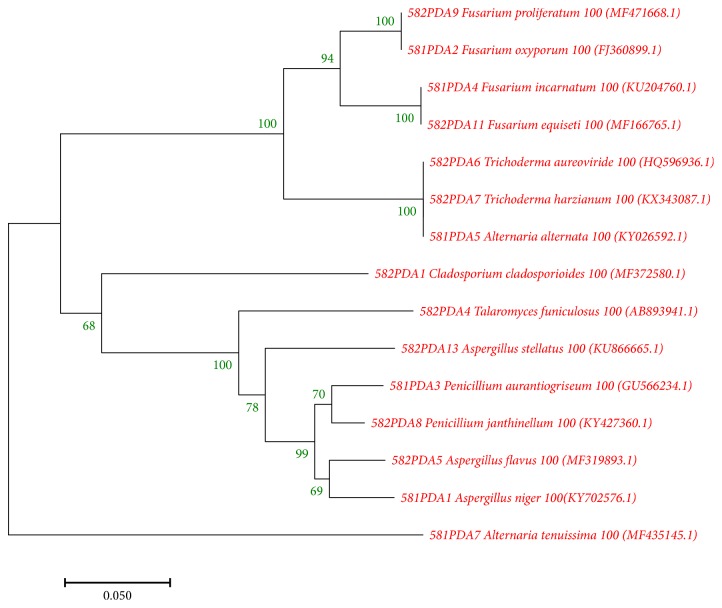
Phylogenetic tree constructed with ITS-rDNA sequences of fungal endophytic isolates obtained from tissue sections of wheat using neighbor-joining method. Evolutionary analyses were conducted in MEGA7.

The need to increase food production and exhaustion of wheat genetic resources has increased interest in alternative approaches for wheat improvement, including the use of endophytes. Previous studies of wheat endophytes showed that all wheat cultivars contain a relatively wide range of endophytes, predominantly fungi [56, 57]. By inspiring with the previous research, current investigation was designed to isolate and characterize endophytic fungi associated with wheat, as well as screening their potentiality to support plant growth through polyphasic approach. Sixteen endophytic fungi were isolated from leaf, stem, and root of different wheat plant samples and were identified using their ITS-rDNA sequences and based on their sequencing result we have chosen 15 isolates for our work (Table 1). The sequences of close relatives were obtained from Gen Bank to reconstruct the phylogenetic tree (Figure 2). After identification, all isolates were screened for PGP and stress tolerance properties and antibiotic sensitivity. The majority of the isolated fungus exhibited a number of relevant growth promoting parameters, including different enzyme activity (ACCD, urease and catalase), inorganic phosphate solubilization; IAA, HCN, siderophore and NH_3_ production (Table 2). They also showed a variable level of resistance against different abiotic stresses and tested antibiotics.

Scientists have proved that ACCD-containing PGPFs can successfully protect against growth inhibition by flooding, elevated salt, drought, and presence of fungal and bacterial pathogens, nematode damage; and existence of high levels of metals and organic contaminants, as well as low temperature stress. In the contemporary investigation, among the 15 isolated fungi 11 were able to show negligible ACCD activity (0.03±0.011 to 1.43±0.01*µ*mol *α*-KB mg^−1^ protein hr^−1^). Previous document showed that a squat level of ACCD activity, such as or more than 0.02*µ*mol *α*-KB mg^−1^ protein hr^−1^ is adequate for a microbe to elevate plant growth as a PGPE [30]. IAA plays a role in cell growth, slows down the growth of side shoots, promotes abscission, forms xylem and phloem tissue, and also affects the growth and elongation of roots [57]. Among the tested fungi, 9 were able to produce IAA with isolate 582PDA4 being the top producer (Table 2). Based on above grounds, we may state that, for the investigated strains, IAA production executes a crucial role in the regulation of this PGP characteristic. Siderophore producing endophytes are beneficial for plants because they inhibit phytopathogens by shrinking the accessibility of iron to pathogens and thus hamper their growth inside the plants and indirectly accelerate the plant growth. We have checked siderophore producing ability of the isolated fungi by universal CAS agar technique and found 3 (581PDA1, 582PDA6, and 582PDA7) isolates having siderophore production ability in the form of orange halo around the colonies (Table 2). Optimistic growth response has been documented in diverse crop plants inoculated with phosphate solubilizing endophytes [58]. Our findings showed that 12 out of 15 tested endophytic fungi gave PSI ranging from 2.11±0.17 to 5.16 ±0.36 (Table 2). HCN and NH_3_ have indirect effect on growth promotion of plants. HCN is volatile in nature and competent to reveal antifungal action whereas NH_3_ can assist to assure the nitrogen requirement of the host plant and in large amount suppresses the colonization of plants by pathogens [59]. HCN test was positive for only 2 (582PDA1 and 582PDA11) isolates, while NH_3_ was produced by around 34% of the tested microbes (Table 2). For urease and catalase tests, we have observed that none of the isolates possess urease enzyme but all of them exhibited positive response for catalase enzyme (Table 2). Catalase enzyme leads a foremost task in organism protection against toxic free radicals that are produced predominantly beneath environmental, mechanical, and chemical stresses and could promote plant growth via an indirect way [60]. In the current investigation all fungi gave positive response for catalase enzyme; hence, we can say they indirectly enhance plant growth. These findings are in agreement with those published previously [61].

It is well known that abiotic stress leads to a series of morphological, physiological, biochemical, and molecular changes that adversely affect plant growth and productivity [62]. Therefore, selection, screening, and application of stress tolerant PGPF for better farming would considerably facilitate the farming community by overcoming such extreme climate changes. Additionally, such microbial application is also acknowledged to conquer the fatal effect of chemical fertilizers and pesticides. Therefore, with growth promoting activities screening we have also taken initiative to drag out and be acquainted with promising wheat endophytic fungi with abiotic stress tolerance and antibiotic sensitivity for better plant growth promotion.

Salt tolerant microbes are a prospective bioresource for saline prone areas. On the other hand, previous research showed if these endophytes also possess plant growth promoting traits, they would be ideal for use in sustainable agriculture [63]. Out of the 15 fungal isolates of wheat plants, 13.33% exhibited tolerance to high salt concentration (10% NaCl). Heavy metal contamination in the environment has turned into a severe issue because they are not degradable like organic pollutants and accumulate in different parts of the food chain which is a threat to plants and animal health. In this perspective, previous research gave information of diverse endophytes having ability to trim down the stress posed on plants by the presence of heavy metals, amplify the accessibility of metal for plant uptake, and promote plant growth [64, 65]. In our current study we have also found a number of wheat endophytic fungi exhibiting resistance towards the tested heavy metal salts (Ni, Cu, Cd, Co, and Pb) in a variable range along with their PGP properties. Crop plant-associated microbes having good drought tolerance property are recently getting increased attention. By influencing plant morphology, development, and physiological and biochemical responses to stress, fungal endophytes can provoke mechanisms of drought escaping, drought lenience, and drought recovery in their hosts [66, 67]. In our investigation, we have observed that all the selected wheat endophytic fungi are able to resist drought in variable range. A number of endophytes have been studied that help plants to cope up with temperature stress and also encourage growth promotion of diverse crops at different climates [68]. We have studied effect of different temperature on all the tested fungal isolates and found the optimum growth temperature of maximum strains is 25°C. The least and utmost temperature tolerated by the isolates were recorded as 5°C (581PDA1) and 55°C (582PDA4), respectively. So it is tempting to conclude that these microbes can help to tolerate temperature stress to certain extent.

Uses of different types of materials such as heavy metals along with antibiotics in plants generate a selective pressure in the environment that consequently leads to the mutation in organism which will help them to survive and multiply [69]. Previous research showed that antibiotic resistance property of endophytes can accelerate plant growth [34, 35, 53]. With this deliberation, the antibiotic resistance among PGPF was checked and we noticed their resistance pattern varied from antibiotic to antibiotic. It has also been reported that under environmental conditions of metal stress, metal and antibiotic resistant microorganisms will adapt faster by the spread of R-factors than by mutation and natural selection. The discrepancy in the resistance to many tested antibiotics probably due to the variation in growth conditions and exposure of PGP microbes to stress conditions or toxic stuffs as well as existence or nonexistence of resistance mechanisms that could be encoded either by chromosome and/or R-plasmid [68, 69].

## 5. Conclusion

The present research revealed that wheat plant is an ecological niche for different putative fungal endophytes. The plant growth promoting ability of these microbes may be due to their capability to secret elevated amounts of various favorable growth promoting metabolites and therefore assist their host plants to survive beneath stress condition. The findings of this study motivate us to advance investigation on the selected fungal endophytes in order to develop a strapping Bioagent with spacious applicability to multifield and hereafter emerge as a thriving bioinoculum leading on the way to organic food crops for a better tomorrow by plummeting the extreme uses of chemicals.

## Figures and Tables

**Table 1:** List of unique isolates from wheat plant and identification of the most closely related species using the ITS sequence to perform an nr/nt BLAST search at the National Center for Biotechnology Information.

Strain	Homologous microorganism	(% Identity)	Accession no.
581PDA1	*Aspergillus niger*	100	KY702576.1
581PDA2	*Fusarium oxyporum*	100	FJ360899.1
581PDA3	*Penicillium aurantiogriseum*	100	GU566234.1
581PDA4	*Fusarium incarnatum*	100	KU204760.1
581PDA5	*Alternaria alternata*	100	KY026592.1
581PDA7	*Alternaria tenuissima*	100	MF435145.1
582PDA1	*Cladosporium cladosporioides*	100	MF372580.1
582PDA4	*Talaromyces funiculosus*	100	AB893941.1
582PDA5	*Aspergillus flavus*	100	MF319893.1
582PDA6	*Trichoderma aureoviride*	100	HQ596936.1
582PDA7	*Trichoderma harzianum*	100	KX343087.1
582PDA8	*Penicillium janthinellum*	100	KY427360.1
582PDA9	*Fusarium proliferatum*	100	MF471668.1
582PDA11	*Fusarium equiseti*	100	MF166765.1
582PDA13	*Aspergillus stellatus*	100	KU866665.1

**Table 2:** PGP traits of isolated endophytic fungi.

Strain	*α* KB *µ* mol mg^−1^ protein hr^−1^	IAA *µ*gml^−1^	Siderophore production	(PSI)	NH_3_ production	HCN	Urease	Catalase
581PDA1	0.99±0.005	-	+	2.64±0.34	++	-	-	++
581PDA2	-	16.5±0.005	-	-	-	-	-	+
581PDA3	0.54±0.015	-	-	2.64±0.34	-	-	-	+
581PDA4	-	-	-	3.13±0.37	-	-	-	+
581PDA5	0.61±0.02	-	-	2.11±0.17	-	-	-	+
581PDA7	0.73±0.015	2.62±0.14	-	2.08±0.03	-	-	-	+
582PDA1	0.99±0.01	-	-	-	-	+	-	+
582PDA4	-	36.12±0.004	-	2.29±0.19	+	-	-	+
582PDA5	0.57±0.005	4.00±0.003	-	-	+++	-	-	++
582PDA6	1.41±0.005	1.125±0.04	++	4.09±0.22	++	-	-	++
582PDA7	1.43±0.01	2.12±0.05	++	2.49±0.16	-	-	-	+
582PDA8	0.64±0.01	5.50±0.007	-	5.16±0.36	-	-	-	+
582PDA9	0.03±0.011	1.375±0.018	-	2.18±0.13	+	-	-	+
582PDA11	-	21.125±0.009	-	2.49±0.17	-	+	-	+
582PDA13	0.89±0.01	-	-	2.17±0.02	+	-	-	+

- = negative; + = poor growth; ++ = moderate growth; +++ = excellent growth. Values represent mean of triplicate readings ±SD.

**Table 3:** Salt tolerance property of the endophytic fungi.

Strain	2.5%	5%	7.5%	10%
581PDA1	+	+	+	-
581PDA2	+	+	+	-
581PDA3	+	+	+	-
581PDA4	+	+	+	-
581PDA5	+	+	+	-
581PDA7	+	+	+	-
582PDA1	+	+	-	-
582PDA4	+	+	-	-
582PDA5	+	-	-	-
582PDA6	+	+	+	+
582PDA7	+	+	+	+
582PDA8	+	+	+	-
582PDA9	+	+	+	-
582PDA11	+	-	-	-
582PDA13	+	+	-	-

Here, + = growth; - = no growth.

**Table 4:** Growth of the tested endophytes in different concentrations of heavy metals amended agar plates.

*Strain*	*Nickel*						*Copper*						*Cadmium*						*Cobalt*						*Lead*					
	50	100	150	200	250	300	50	100	150	200	250	300	50	100	150	200	250	300	50	100	150	200	250	300	50	100	150	200	250	300
581PDA1	+	+	+	+	-	-	+	+	+	+	+	+	+	+	+	+	-	-	+	+	+	+	-	-	+	+	+	+	+	+
581PDA2	+	+	+	+	-	-	+	+	+	+	-	-	+	+	+	+	-	-	+	+	+	+	-	-	+	+	+	+	-	-
581PDA3	+	+	+	+	-	-	+	+	+	+	-	-	+	+	+	+	-	-	+	+	+	+	-	-	+	+	+	+	-	-
581PDA4	+	+	+	+	-	-	+	+	+	+	+	-	+	+	+	+	-	-	+	+	+	+	-	-	+	+	+	+	+	-
581PDA5	+	+	+	+	-	-	+	+	+	+	-	-	+	+	+	+	-	-	+	+	+	+	-	-	+	+	+	+	-	-
581PDA7	+	+	+	+	-	-	+	+	+	+	+	+	+	+	+	+	-	-	+	+	+	+	-	-	+	+	+	+	+	+
582PDA1	+	+	+	+	+	-	+	+	+	+	+	+	+	+	+	+	-	-	+	+	+	+	+	-	+	+	+	+	+	+
582PDA4	+	+	+	+	-	-	+	+	+	+	-	-	+	+	+	+	-	-	+	+	+	+	-	-	+	+	+	+	-	-
582PDA5	+	+	+	+	-	-	+	+	+	+	-	-	+	+	+	+	-	-	+	+	+	+	-	-	+	+	+	+	-	-
582PDA6	+	+	+	+	+	+	+	+	+	+	+	+	+	+	+	+	+	+	+	+	+	+	+	+	+	+	+	+	+	+
582PDA7	+	+	+	+	+	+	+	+	+	+	+	+	+	+	+	+	+	+	+	+	+	+	+	+	+	+	+	+	+	+
582PDA8	+	+	+	+	-	-	+	+	+	+	-	-	+	+	+	+	-	-	+	+	+	+	-	-	+	+	+	+	+	-
582PDA9	+	+	+	+	+	+	+	+	+	+	+	-	+	+	+	+	+	-	+	+	+	+	+	-	+	+	+	+	+	-
582PDA11	+	+	+	+	-	-	+	+	+	+	+	-	+	+	+	+	+	-	+	+	+	+	+	-	+	+	+	+	+	-
582PDA13	+	+	+	+	-	-	+	+	+	+	+	+	+	+	+	+	+	-	+	+	+	+	+	-	+	+	+	+	+	+

Here, + denotes growth; - denotes no growth.

**Table 5:** Drought resistance property of the isolated fungi at different concentrations of PEG.

Strain	10%	20%	30%	35%	40%
581PDA1	34.91	26.72	18.04	4.52	-
581PDA2	73.33	63.67	54.67	7.0	-
581PDA3	66.56	49.30	13.41	-	-
581PDA4	59.78	48.88	4.69	-	-
581PDA5	71.58	54.51	39.97	7.63	-
581PDA7	68.47	42.48	23.56	14.49	-
582PDA1	29.47	8.98	-	-	-
582PDA4	40.64	18.39	6.72	-	-
582PDA5	52.61	35.60	7.8	-	-
582PDA6	84.15	69.79	54.59	35.68	-
582PDA7	78.04	67.59	55.63	46.56	-
582PDA8	39.57	11.96	22.78	-	-
582PDA9	39.81	33.44	16.73	-	-
582PDA11	37.79	12.45	5.19	-	-
582PDA13	37.23	12.13	3.23	-	-

Here, - denotes no growth.

**Table 6:** Growth of the endophytic fungi at different temperatures.

Strain	5°C	15°C	25°C	35°C	45°C	50°C	55°C
581PDA1	+	++	+++	+++	+	+	–
581PDA2	-	+	+++	++	+	–	–
581PDA3	–	+	+++	++	+	–	–
581PDA4	–	+	+++	++	+	–	–
581PDA5	-	++	+++	++	+	+	–
581PDA7	-	++	+++	++	+	+	–
582PDA1	+	++	+++	+++	–	–	–
582PDA4	-	++	+++	++	+	+	+
582PDA5	-	++	+++	++	+	+	–
582PDA6	–	++	+++	++	+	–	–
582PDA7	–	++	+++	++	+	–	–
582PDA8	–	++	+++	++	+	–	–
582PDA9	–	++	+++	++	+	–	–
582PDA11	–	++	+++	++	++	+	–
582PDA13	–	++	+++	++	++	+	–

Here, - = no growth; + = poor growth; ++ = moderate growth; +++ = excellent growth.

**Table 7:** Antibiotic sensitivity of endophytic fungi of *T. aestivum*.

Strain	Nystatin	Ketoconazole	Itraconazole
581PDA1	+	–	–
581PDA2	-	–	–
581PDA3	+	+	+
581PDA4	+	+	+
581PDA5	-	–	–
581PDA7	-	–	–
582PDA1	-	+	+
582PDA4	+++	–	–
582PDA5	+	–	–
582PDA6	+	+	+
582PDA7	+	+	+
582PDA8	-	+	+
582PDA9	+	–	–
582PDA11	-	+	+
582PDA13	-	++	++

Here, - = sensitive to antibiotic; + = poorly resistant; ++ = moderately resistant; +++ = highly resistant.

## Data Availability

The data used to support the findings of this study are available from the corresponding author upon request.
